# Machine learning framework for depression subtype grouping: integrating high-resolution imaging and clinical symptom analysis via correlation and clustering

**DOI:** 10.3389/fpsyt.2026.1747824

**Published:** 2026-03-24

**Authors:** Gaurav Verma, Yael Jacob, Laurel S. Morris, Xin Xing, Bradley N. Delman, James W. Murrough, Ai-Ling Lin, Priti Balchandani

**Affiliations:** 1 Department of Radiology, Icahn School of Medicine at Mount Sinai, New York, NY, United States; 2 Department of Psychiatry, Icahn School of Medicine at Mount Sinai, New York, NY, United States; 3 Department of Computer Science, University of Nebraska Omaha, Omaha, NE, United States; 4 Depression and Anxiety Center for Discovery and Treatment, Department of Psychiatry, Icahn School of Medicine of Mount Sinai, New York, NY, United States; 5 Nash Family Department of Neuroscience, Icahn School of Medicine at Mount Sinai, New York, NY, United States; 6 VISN 2 Mental Illness Research, Education, and Clinical Center (MIRECC), James J. Peters VA Medical Center, Bronx, NY, United States; 7 Department of Radiology, Division of Biological Sciences, and Institute for Data Science and Informatics, University of Missouri, Columbia, MO, United States

**Keywords:** biological subtypes, canonical correlation analysis (CCA), childhood trauma questionnaire (CTQ), FreeSurfer, hierarchical clustering, machine learning based classification, major depression disorder (MDD), ultrahigh field (UHF) magnetic resonance imaging (MRI)

## Abstract

**Introduction:**

Major depressive disorder (MDD) affects approximately one in six individuals over their lifetime, with many patients experiencing treatment-resistant depression, characterized by inadequate or insufficient response from at least one antidepressant treatment. Current classification strategies for depression rely primarily on clinical assessment of symptom severity, which are prone to reader bias and test-retest variability. Moreover, these symptom-based subtypes have shown limited utility in predicting treatment response. This study introduces a data-driven, non-biased classification framework that integrates clinical features with high-resolution magnetic resonance imaging (MRI)-derived features. Using canonical correlation analysis (CCA) and hierarchical clustering, the approach identifies distinct subtypes of MDD, offering a more objective and potentially predictive alternative to traditional methods.

**Materials & methods:**

Sixty-four participants with MDD currently experiencing a major depressive episode and not currently undergoing treatment completed a battery of 11 clinical symptom severity assessments and scanned with 7T T1-weighted MRI with parameters: TE/TR = 3.62/6000 ms, 320x240x240 array size with 224x168 mm^2^ field-of-view (FOV) for voxel dimensions of 0.7 mm^3^ isotropic. The images were automatically segmented using the FreeSurfer 6.0 package and 87 resulting imaging features, along with 11 clinical measures were processed through CCA to derive highly-correlated clinical-imaging phenotypes. An analysis using the Sillhouette and other methods determined an optimal number of clusters for this dataset. Participants with MDD were plotted on axes consisting of highly correlated clinical-imaging phenotypes derived from CCA and subsequently grouped through hierarchical clustering.

**Results:**

CCA identified three highly correlated (r > 0.9) clinical-imaging variable pairs. The first, an anhedonia-related phenotype, showed high loadings from anhedonia severity and brainstem features. The second phenotype was associated with childhood trauma and anhedonia, with the right frontal pole as the primary imaging feature. The third phenotype linked general distress and perceived stress with the right superior temporal lobe. Hierarchical clustering along these canonical axes revealed two distinct clusters: one characterized by high childhood trauma scores and the other showing scores comparable to healthy controls.

**Conclusion:**

Taken together, this study presents a novel ML framework for classifying depression using CCA and clustering.

## Introduction

Major Depressive Disorder (MDD) is a debilitating illness and associated with feelings of sadness or apathy, markedly increased risk for suicide and disturbances in sleep and appetite. MDD affects roughly one in six people over a lifetime with approximately one in fifteen adults (6.7%) experiencing depression in a given year (1, 2). Women are approximately twice as likely as men to experience depression (3), and depression remains among the largest contributors to the global economic burden among all diseases (4). Unfortunately, more than half (50-60%) of afflicted patients experience treatment-resistant depression (TRD), meaning they experience inadequate or insufficient response from one or more antidepressant treatments (5). While the underlying mechanisms of treatment resistance are not fully-understood, medical comorbidities and heterogeneity in depressive severity or subtype may be contributing factors (6). Previous research has focused on characterizing depression according to patterns or severity of symptoms through clinical assessment. Clinical subtyping of depression is subject to test-retest variability both from individual responses and inter-rater bias. Further, patients with distinct etiology may present with similar symptoms and other clinical characteristics, confounding the predictive capacity of these measures (7). Consequently, these clinical markers have not proven reliable in predicting or guiding individual treatment response. This limitation underscores the need to develop data-driven and reproducible biomarkers for MDD and better understand the neurobiological mechanisms underpinning the psychological dysfunction in the brain.

Magnetic Resonance Imaging (MRI) is a non-invasive technique for characterizing neuroanatomy and neurophysiology. Because the signal quality and therefore imaging resolution of MRI is directly proportional to magnetic field strength, scanning at the ultrahigh field strength of 7T may facilitate more accurate visualization of small imaging features compared to the more prevalent clinical field strengths of 1.5T and 3T (8–10). Specifically, better contrast between cortical and subcortical volumes and more precise measurements of structural volumes may enable more reliable quantification of anatomical structures. Combined with automated whole-brain segmentation and data-driven, machine-learning based classification algorithms, 7T MRI can provide accurate and reliable information about subtle cortical and subcortical morphological changes in depression patients.

The presented study aims to implement machine learning analysis to stratify depression patients according to both clinical and imaging-derived features and classify them along these dimensions using hierarchical clustering. Participants with MDD were clustered along their data-derived canonical variables, and the clinical characteristics of participants along these clusters were then probed. This study leverages the high resolution and image quality of 7T MRI to investigate how imaging features correlate with clinical measures and how both relate to treatment response.

## Materials & methods

**Figure 1:**
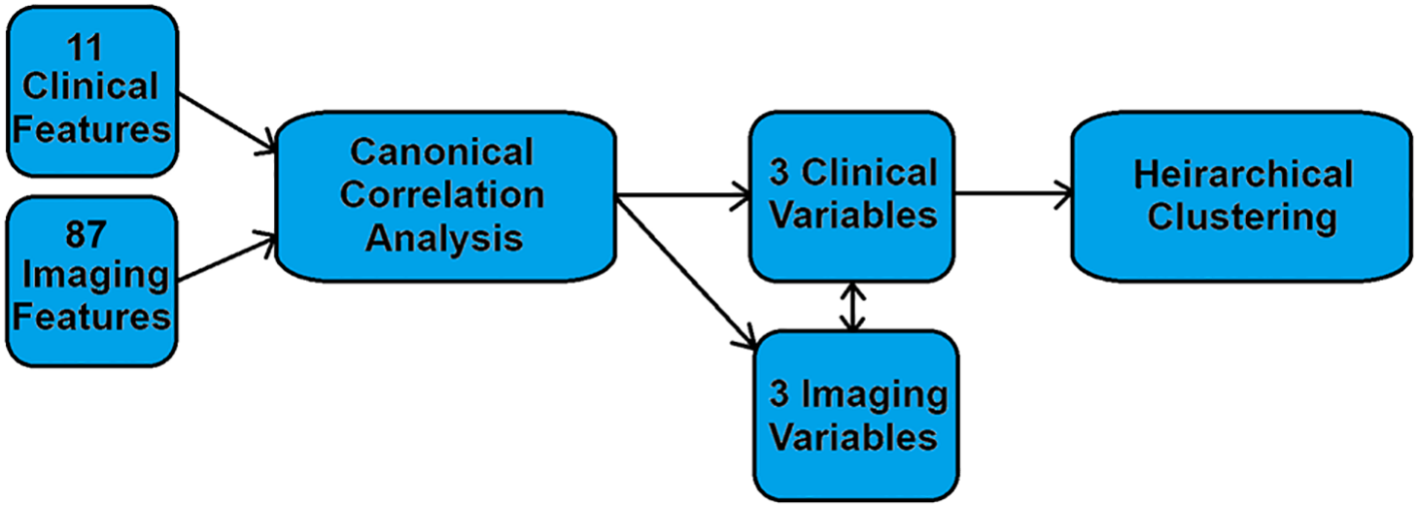
Flow chart showing the analysis scheme.

**Figure 1** shows the flow chart of the study. Eleven clinical features and 87 imaging-derived features are combined using canonical correlation, generating three highly-correlated pairs of clinical and imaging variables. The clinical variables are then clustered using hierarchical clustering, although clustering on the imaging-derived variables would yield highly similar results due to their high correlation.

### Clinical and imaging data acquisition

Following informed consent, study participants were evaluated based on a structure interview by trained clinicians and depression severity was measured using the clinician administered Montgomery-Åsberg Depression Rating Scale (MADRS) (11). Individuals taking antidepressant or other psychotropic medication or using recreational drugs at the time of the study were excluded. Individuals with ferromagnetic implants or for whom MRI was otherwise contraindicated were also excluded. Sixty-four participants in a current major depressive episode (MDE) (32 female, 32 male, age 32.8 ± 10.4 years) and sixty-seven healthy controls (32 female, 35 male, age 34.5 ± 10.1 years) were enrolled and scanned on a Siemens Magnetom 7T whole-body MRI scanner (Siemens Healthineers, Erlangen, Germany) with a 32Rx/1Tx channel Nova head coil (Nova Medical, Wilmington, MA). The primary anatomical imaging study was T_1_-weighted magnetization rapid gradient-echo (MPRAGE) sequence with the following parameters: echo time (TE) = 3.62 ms, repetition time (TR) = 6000 ms, field-of-view (FOV) = 224 x 168 mm^3^, array size = 320 x 240, slices = 240, voxel size = 0.7 mm^3^ isotropic, acquisition time = 7:26 minutes. All participants were recruited at the Depression and Anxiety Center for Discovery and Treatment (DAC) at the Icahn School of Medicine at Mount Sinai between 2017 and 2023 and all data were collected after obtaining informed written consent as approved by the Institutional Review Board (IRB). Prospective participants underwent Structured Clinical Interview for DSM-V Axis Disorders (SCID-V) by a trained rater to determinepsychiatric status. Exclusion criteria included being younger than 18 years old, presence of metallic implants or other contraindications to MRI, history of schizophrenia or other psychotic disorder and current use of recreational drugs or anti-depressant medications. Patients were not asked to discontinue or delay current treatment, but were excluded from recruitment for the study to avoid the potential confounding effects of their medication on clinical or neuroimaging measures. All scans were performed under a protocol approved by the local institutional review board (IRB) and imaging data were stripped of private health information (PHI) in compliance with Health Information Portability and Accountability Act (HIPAA).

In addition to the MADRS, clinical questionnaire data included the Mood and Anxiety Symptom Questionnaire – General Distress (MASQ GD), Anhedonic Depression (MASQ AD) and Anxious Arousal (MASQ AA) (12), the Perceived Stress Scale (PSS) (13), the Childhood Trauma Questionnaire (CTQ) (14), the Ruminative Response Scale (RRS) (15), the Snaith-Hamilton Pleasure Scale (SHAPS) (16), the Temporal Experience of Pleasure Scale – Anticipatory (TEPS-A) and Consummatory (TEPS-C) (17) and the State-Trait Inventory for Cognitive and Somatic Anxiety (STICSA) (18). Self-reported counts of lifetime of antidepressant treatment failures were recorded for participants with MDD, along with age of onset and duration of current episode. Variations in these metrics were evaluated following the data-driven clustering, though were not included in the correlation analysis or clustering itself.

T1-weighted imaging data were post-processed using the FreeSurfer version 6.0 automatic segmentation algorithm (19), which uses a probabilistic model applying position and imaging contrast to derive volumetric imaging features. In all, 68 cortical regions were evaluated (34 from each hemisphere) along with 19 non-cortical regions – thalamus, caudate, putamen, pallidum, hippocampus, amygdala, ventral diencephalon, choroid plexus and nucleus accumbens from each hemisphere, and total brain stem volume. All volumes were normalized to the total brain volume for each subject, measured as the brain segmentation volume excluding the ventricles (FreeSurfer function *BrainSegVolNotVent*).

### Machine learning analysis

The machine learning analysis was divided into two steps: In part 1, canonical correlation analysis (CCA) was performed on clinical and imaging features derived from a combination of MDD participants and healthy controls to generate highly-correlated clinical-imaging phenotypes (7, 20–22). While controls tended to report lower severity and variability in clinical measures than MDD patients, the combination of both cohorts in the correlation analysis produced a more variable group than either cohort individually. Indeed, canonical correlation performed on the MDD patients alone produced rank errors due to their limited dynamic range compared to the combined cohort. Clinical-imaging variable sets very strong correlation (defined as those with greater than 0.9 correlation) (23) were selected for the second part of the analysis and labeled as clinical-imaging phenotypes. In part 2, hierarchical clustering based clustering of the MDD participants along these data-driven phenotypes. All source code for the analysis can be found at https://github.com/linbrainlab/MDD-project. The canonical correlation and clustering steps were both performed using native functions in Matlab (The Mathworks, Natick, MA).

#### Step 1: canonical correlation analysis

**Figure 2:**
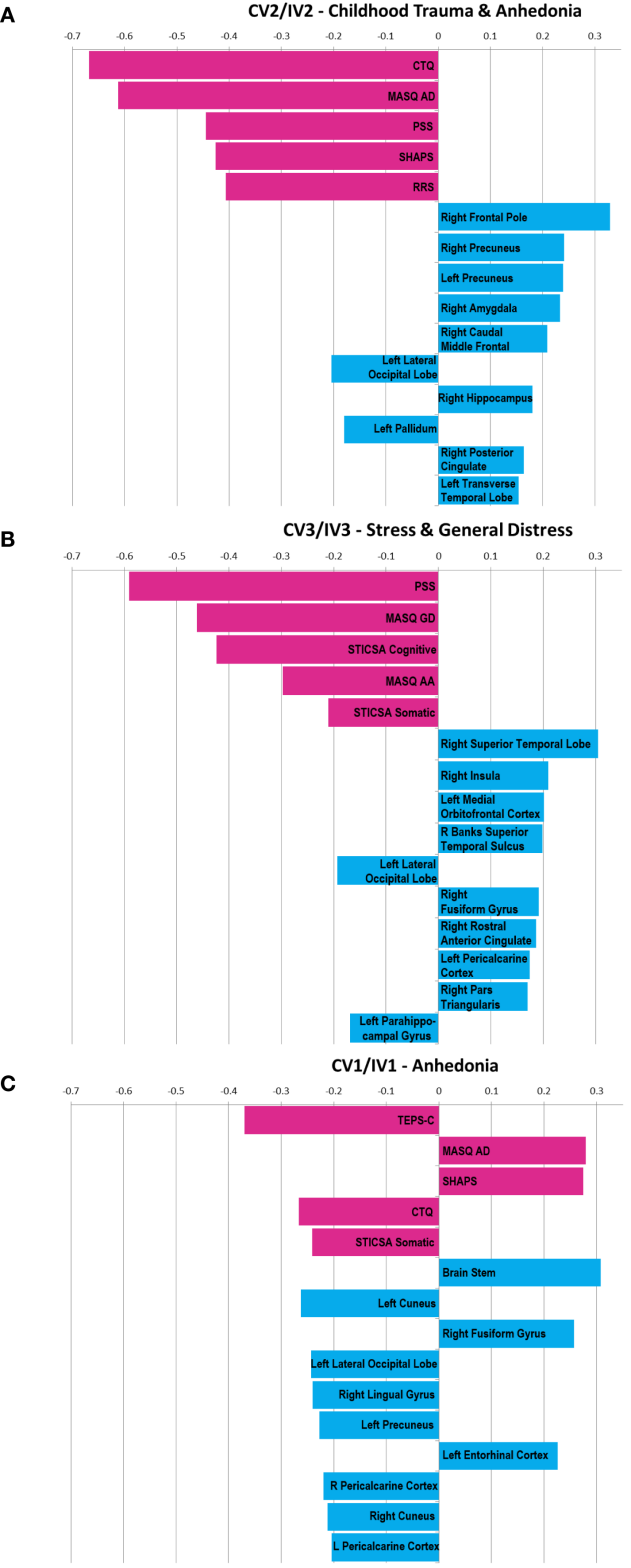
**(A–C)** Bar charts showing clinical components of the canonical variables emerging from the analyzed MDD participant cohort. The first canonical variable (CV 1/IV 1) related to Anhedonia is shown in **(A)**. **(B)** shows the second canonical variable (CV 2/IV 2) related to childhood trauma and anhedonia. **(C)** shows the third canonical variable (CV 3/IV 3) related to stress & general distress. Clinical measures representing the largest loading factors of the variable are shown in pink while the top ten imaging-derived loading factors are shown in blue.

Canonical correlation analysis explores the relationship between distinct multivariate datasets, in this case clinical symptom scales and imaging-derived features, by identifying axes of maximal correlation. A total of 87 (68 cortical and 19 non-cortical) MRI-derived normalized volumetric features were generated by the quantification and segmentation algorithm. These represented the dependent variables of the correlation analysis. Eleven clinical measures were evaluated as the independent variables of the correlation analysis (**Table 1**). The correlation between these measures is seen in **Table 2**. All measures besides TEPS-A and TEPS-C showed positive correlation with each other and were generally higher among MDD patients compared to controls. TEPS-A and TEPS-C were negatively correlated with the other measures and generally lower among MDD patients. CCA generated a series of clinically-derived canonical variables CV 1–11 representing linear combinations of clinical components. CCA generated a corresponding set of imaging-derived canonical variables IV 1–11 representing linear combinations of imaging components. CCA aims to generate pairs of these canonical variables (e.g. CV 1 and IV 1) such that the correlation between them is maximized. Canonical pairs relate their component datasets to one another, and a highly-correlated canonical pair can be understood as an axis for stratifying both clinical and imaging data. Following CCA, the most highly-correlated imaging and clinical canonical variable pairs (defined as r > 0.90) were labeled as clinical-imaging phenotypes and considered for the clustering analysis (**Figure 2**). The input data were normalized by z-score and canonical correlation was performed using the native canoncorr function in Matlab R2021a.

**Table 1:** The eleven clinical measures considered in the canonical correlation analysis.

No.	Clinical metric	Clinical scale name	▲ in MDD
1	MASQ GD (24)	Mood & anxiety symptoms questionnaire (Generalized Distress)	▲
2	MASQ AD	Mood & anxiety symptoms questionnaire (Anhedonic Depression)	▲
3	MASQ AA	Mood & anxiety symptoms questionnaire (Anxious Arousal)	▲
4	PSS (13)	Persistent Stress Score	▲
5	CTQ (14)	Childhood Trauma Questionnaire	▲
6	RRS (15)	Ruminative Response Scale	▲
7	SHAPS (16)	Snaith-Hamilton Pleasure Scale	▲
8	TEPS-A (17)	Temporal Experience of Pleasure Scale (Anticipatory)	∇
9	TEPS-C	Temporal Experience of Pleasure Scale (Consummatory)	∇
10	STICSA C (18)	State-trait inventory for cognitive and somatic anxiety (Cognitive)	▲
11	STICSA S	State-trait inventory for cognitive and somatic anxiety (Somatic)	▲

The final column describes whether the measure tended to be higher (▲) or lower (∇) among total MDD patients analyzed in this study in comparison to the control cohort.

**Table 2:** Correlation of clinical measures among the combined MDD participants and control cohorts.

MASQ GD	1.00										
MASQ AD	0.59	1.00									
MASQ AA	0.61	0.24	1.00								
PSS	0.80	0.62	0.60	1.00							
CTQ	0.55	0.49	0.35	0.53	1.00						
RRS	0.86	0.58	0.60	0.83	0.48	1.00					
SHAPS	0.58	0.57	0.29	0.52	0.44	0.56	1.00				
TEPS-A	-0.54	-0.69	-0.25	-0.51	-0.48	-0.48	-0.61	1.00			
TEPS-C	-0.44	-0.57	-0.21	-0.44	-0.41	-0.43	-0.61	0.77	1.00		
STICSA C	0.86	0.56	0.65	0.83	0.52	0.86	0.53	-0.49	-0.46	1.00	
STICSA S	0.63	0.32	0.85	0.68	0.47	0.67	0.34	-0.33	-0.26	0.73	1.00
	MASQ GD	MASQ AD	MASQ AA	PSS	CTQ	RRS	SHAPS	TEPS-A	TEPS-C	STICSA C	STICSA S

Arrows indicating higher levels in MDD patients were in blue while arrows indicating lower levels in MDD patients were pink. All clinical measures showed positive correlation with each other, except TEPS-A and TEPS C, which were negatively correlated with the others. Clinical measure labels follow the convention outlined in **Table 1**.

#### Step 2: hierarchical clustering

Following the canonical correlation, the subset of canonical variable pairs which produced strong correlation between the clinically-derived and imaging-derived measures were selected for clustering analysis. In the combined MDD patient and control subsets, this process highlighted three such canonical variable pairs: the clinically-derived CV 1–3 and the imaging-derived IV 1-3. MDD patients were plotted on the resulting three-dimensional spaces prescribed by CV 1–3 and hierarchical clustering was performed with two prescribed clusters (C = 2), as optimized in the earlier analysis. These two clusters were then analyzed for differences in clinical and imaging measures, as well as differences in other characteristics like age, gender, age of onset, duration of current episode and antidepressant treatment history. The hierarchical clustering used Ward’s minimum variance method was used to calculate Euclidean distance between clusters. Clustering was performed using the native linkage and cluster functions in Matlab R2021a.

## Results

Canonical correlation analysis produced three canonical variables with high correlation between clinical and imaging derived phenotypes and patients were clustered along the three-dimensional space defined by the three canonical variables CV 1-3. **Figures 2A–C** show the canonical variables generated by step 1 of the study, and their loading from the top five component clinical measures. **Figures 2A–C** also show the ten imaging features with the highest absolute loading from the clinical canonical variables CV1-3. Five clinical features and ten imaging features were chosen as a compromise between figure clarity and comprehensiveness, and the imaging features were correlated to the clinical canonical variables to be consistent with the clinical scales. The loading from each clinical and imaging feature for CV1–3 as well as IV1–3 are included in the **Supplementary Data**. The three highlighted canonical variables, CV 1-3, had correlations of 0.95, 0.92 and 0.91 with their corresponding imaging-derived canonical variables IV 1-3. These three canonical variables will be labeled as Anhedonia-related (CV 1/IV 1), Child Trauma and Anhedonia related (CV 2/IV 2) and Stress and General Distress related (CV 3/IV 3). A clinical interpretation of the canonical variables is listed as follows:

### CV 1/IV 1 - “anhedonia related”

The canonical pair CV 1 and IV 1 showed the highest correlation with each other at r = 0.95, and CV 1 showed high loading from anhedonia-related measures including TEPS-C (r = -0.37), MASQ AD (r = 0.28) and SHAPS (r = 0.27). CV 1/IV 1 also showed negative loading from the childhood trauma measure CTQ (r = 0.27). Patients with high anhedonia and a low childhood trauma score would show a higher CV 1/IV 1. Because some clinical symptom scales are strongly correlated amongst each other, the signs of their loading factor within each canonical variable must be carefully considered to contextualize their clinical characteristics. For example, loading from STICSA-C (r = 0.19) and STICSA-S (r = -0.24) in CV 1 had opposite signs, yet these measures were highly correlated with each other at r = 0.73 among the 131 total subjects analyzed. Their combination within CV 1 thus represents minimal contribution from anxiety-related measures, in agreement with the low loading from the other anxiety-related measure, MASQ AA, at r = -0.01.

### CV 2/IV 2 - “childhood trauma and anhedonia related”

CV 2/IV 2 showed highest loading from the CTQ (r = -0.67) and MASQ AD measures (r = -0.61), and can be described as a childhood trauma & anhedonia-related clinical variable. Compared to CV 1/IV 1, the contribution from anhedonia and childhood trauma measures was greater, and in the same direction. That is, patients with low childhood trauma measures and *low* anhedonia-related measures would show a higher CV 2/IV 2. CV 2/IV 2 also much stronger loading from stress and anxiety related measures than CV 1/IV 1 with negative loadings from MASQ GD (r = -0.35), MASQ AA (r = -0.35), STICSA-C (r = -0.26) and STICSA-S (r = -0.27).

### CV 3/IV 3 - “stress and general distress related”

CV 3 showed highest loading from PSS (r = -0.59) and MASQ GD (r = -0.46) measures as well as STICSA-C (r = -0.42), and can be described as a stress & anxiety-related clinical variable. In contrast to the other two canonical pairs, CV 3/IV 3 showed minimal loading from the anhedonia-related clinical measures MASQ AD (r = -0.08), SHAPS (r = 0.01), TEPS-A (r = 0.10) and TEPS-C (r = 0.09).

**Figure 3:**
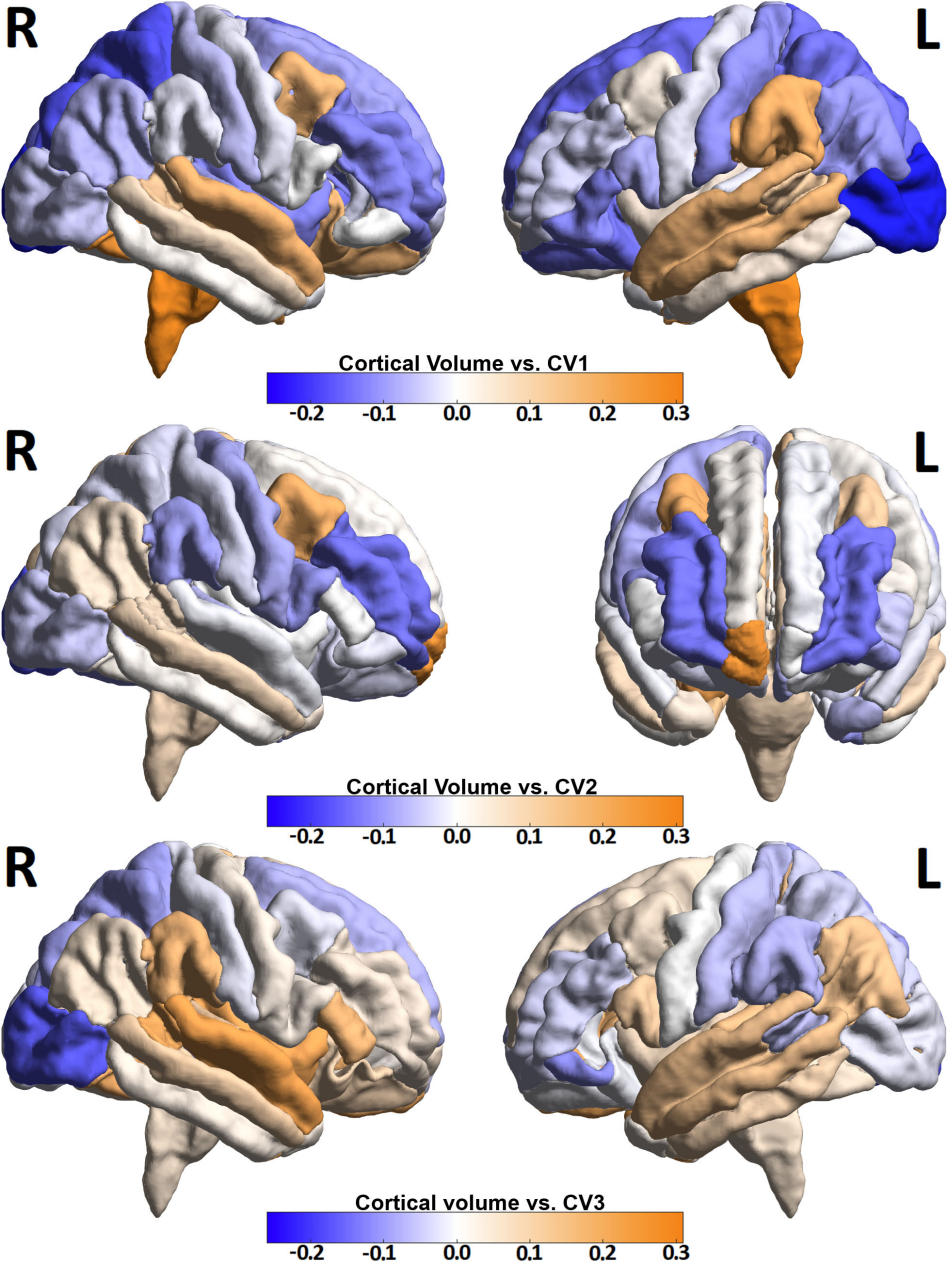
Three volumetric heat maps identifying cortical regions with highest correlations with CV 1-3. Deeper blue colors represent negative correlations, down to -0.26, and deeper orange colors represent positive correlations, up to 0.31. The images are generally projected in sagittal orientation to show left and right hemispheres except for the middle set (correlations with CV 2), which shows anterior projection instead of left lateral to emphasize the maximum positive correlation from the right frontal pole.

**Figure 4:**
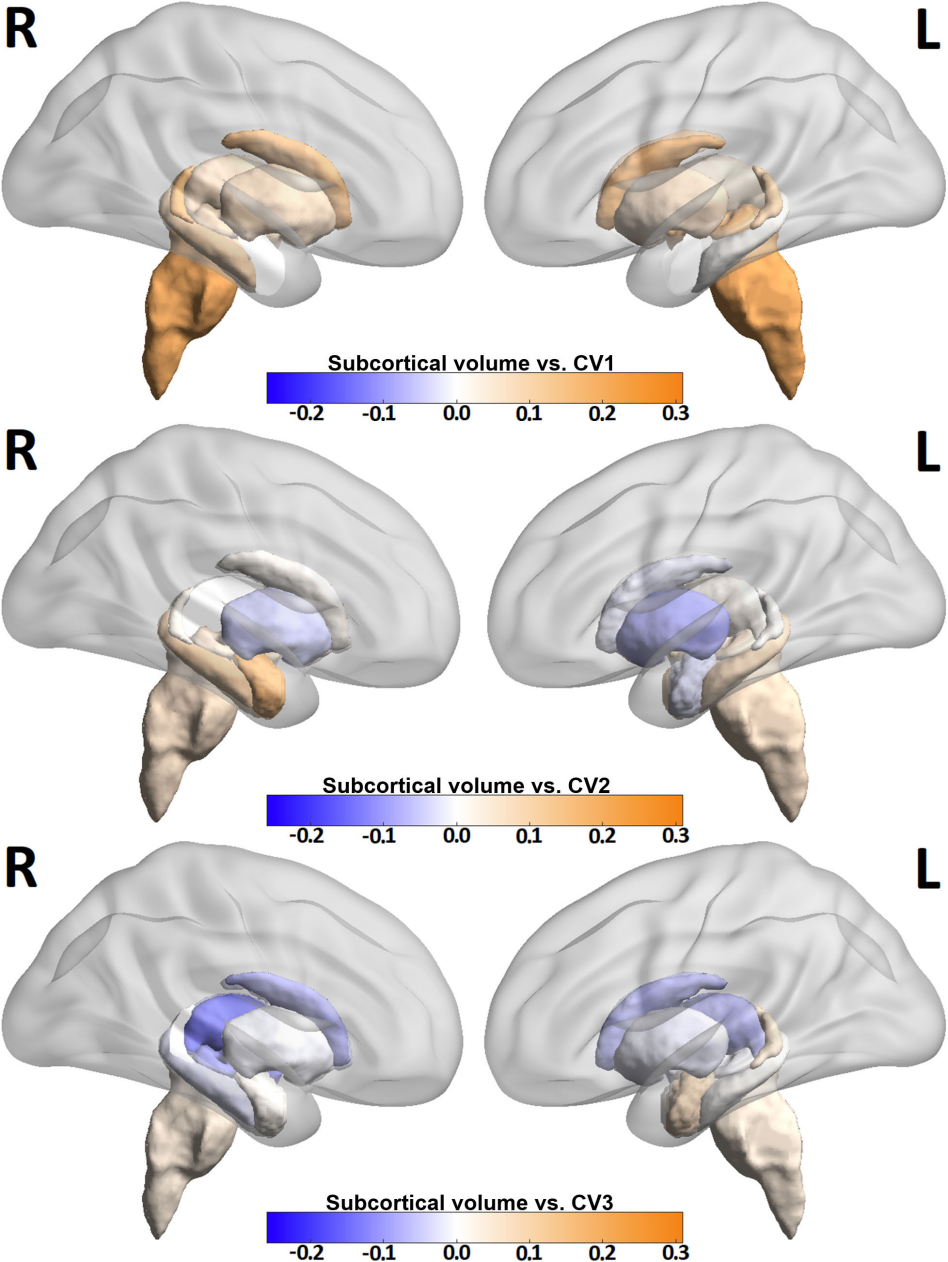
Three volumetric heat maps identifying subcortical regions with highest correlations with CV 1-3. Deeper blue colors represent negative correlations, down to -0.26, and deeper orange colors represent positive correlations, up to 0.31. The images are projected in sagittal orientation showing left and right hemispheres.

**Figures 3** and **4** show heat maps depicting correlations between cortical (**Figure 3**) and sub-cortical (**Figure 4**) imaging metrics and the derived canonical variables CV 1-3. CV 1 showed greatest loading from brain stem volume (r = 0.31) as well as contributions from left (r = 0.26) and right cuneus (r = 0.21). CV 2 showed greatest loading from right frontal pole (r = 0.30), right precuneus (r = 0.22) and left precuneus (r = 0.22). Among sub-cortical regions, CV 2 showed greatest loading from right Amygdala (r = 0.21). CV 3 showed greatest loading from right superior temporal lobe (r = 0.28). The top ten imaging metrics contributing to CV 1–3 are shown in blue at the bottom of **Figures 2A–C**.

In order to determine the optimal number of clusters, we used a combination of metrics used to assess the internal validity of the clusters (**Table 3**). The silhouette score is an aggregate score that determines the similarity of each point to the cluster they have been assigned, as compared to all other clusters (25); the Calinski-Harabaz score evaluates the compactness of a cluster as a function of the within-cluster dispersion and the between-cluster dispersion (26); lastly, the Davies Bouldin index estimates goodness of cluster assignment as the average similarity of each point, defined as the ratio of within-cluster distances to between cluster distances (27). Combined, these metrics can indicate the most statistically optimal number of clusters to use (28, 29). Based on optimization using these analyses, the machine learning clustering strategies were used to generate two clusters during the hierarchical clustering step.

**Table 3:** Analysis to determine optimal number of clusters in the MDD participant cohort, as determined by Calinski-Harabasz, Davies-Bouldin and Silhouette measures.

Clusters	Calinski-Harabasz	Davies-Bouldin	Silhouette
2	12.4817	2.0356	0.2813
3	11.3004	1.9282	0.2286
4	9.8071	1.7995	0.2216
5	9.0195	1.8122	0.2067
6	8.4541	1.6756	0.2132
7	8.1465	1.6607	0.2009
8	7.7400	1.5165	0.1858

Based on these results, an optimal number of two clusters was selected.

**Figure 5:**
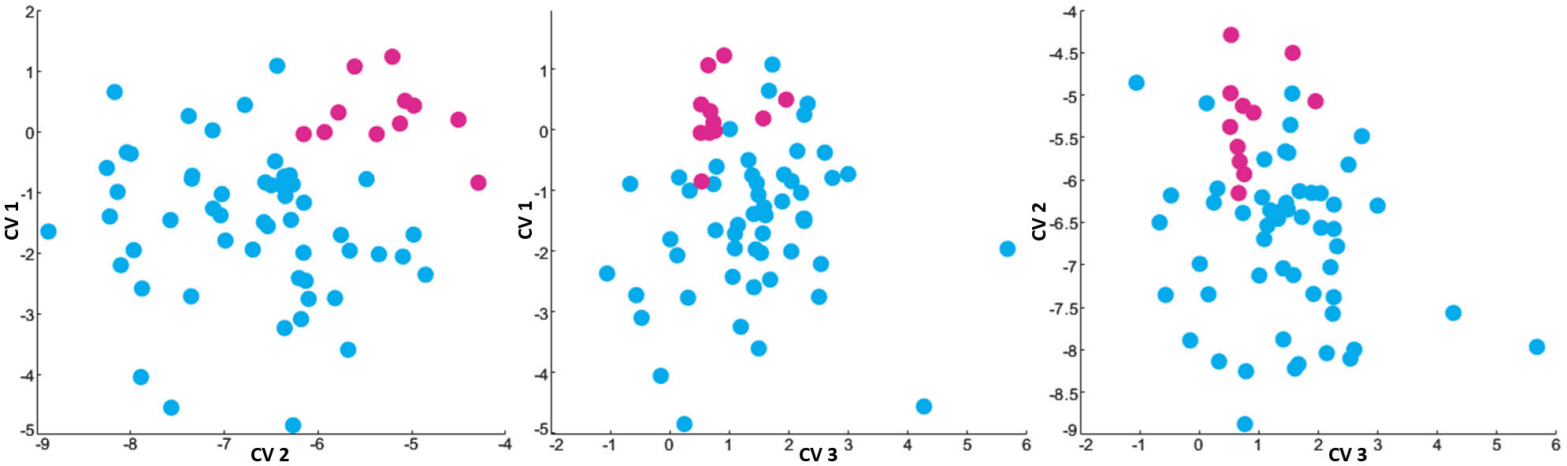
Hierarchical clustering results (C = 2, indicating two prescribed clusters) of MDD patients in the three-dimensional space generated by plotting subjects along CV 1-3. MDD Cluster CL 1 is shown in light blue while MDD cluster CL 2 is shown in dark pink. The three-dimensional space delineated by CV 1–3 is presented in three sub-figures: CV 1 vs. CV 2, CV 1 vs. CV 3 and CV 2 vs. CV 3, respectively from left to right.

Hierarchical clustering along the canonical variables CV 1–3 produced two clusters as shown in **Table 4** and plotted on axes representing the canonical variables in **Figure 5**. Clustering divided the 64 patients in the MDD cohort into clusters of 53 patients (CL 1, 33.6 ± 11.0 years, with 27 men and 26 women) and 11 patients (CL 2, average age 28.5 ± 5.4 years, containing 5 men and 6 women). CL 2 patients were characterized by higher childhood trauma scores (CTQ) than CL 1 patients, whereas CL 1 patients showed CTQ scores not significantly different than healthy controls. CL 2 patients also showed significantly lower STICSA Cognitive scores than CL 1. As shown in **Table 4**, no significant differences were seen between age, gender composition, composite MADRS score or BMI between the two patient clusters. As shown in **Table 5**, the two clusters showed significantly different values in CV1, CV2, IV1 and IV2 with CV3 and IV3 trending towards significance. The corresponding **Figure 5** shows scatterplots showing the distribution of the patient cohort along axes representing CV 1-3. CL 1 is shown in light blue and CL 2 is shown in a darker pink.

**Table 4:** Averages for healthy controls, cluster CL 1 and cluster CL 2 along with results for two-sided student’s t-test comparisons between those groups for demographic characteristics and clinical symptom scales.

7TID	HC (n=67)	CL1 (n=53)	CL2 (n=11)	CL1 vs. CL2	HC vs. CL1	HC vs. CL2
Age (years)	35 ± 10	28 ± 5.4	34 ± 11	1.3E-01	5.6E-02	6.6E-01
Gender (% Female)	0.48	0.55	0.49	7.5E-01	6.8E-01	8.9E-01
Height (Inches)	67 ± 3.7	68 ± 5.4	68 ± 3.9	9.3E-01	4.9E-01	2.6E-01
Weight (Pounds)	164 ± 38	160 ± 29	165 ± 42	7.7E-01	8.1E-01	8.7E-01
BMI	25 ± 5.3	24 ± 5.0	25 ± 5.3	7.9E-01	6.3E-01	7.0E-01
MDD Onset (years)		18	21	6.0E-01		
MDD Duration (months)		55	59	9.0E-01		
Treatment Failures		1.3 ± 1.4	1.8 ± 2.3	6.8E-01		
MADRS	0.6 ± 1.1	27.9 ± 7.7	30.0 ± 4.8	4.2E-01	2.9E-56*	2.4E-55*
MASQ GD	12 ± 3.1	28 ± 7.8	30 ± 8.7	5.2E-01	7.4E-19*	1.2E-29*
MASQ AD	31 ± 8.4	43 ± 6.7	45 ± 5.9	3.6E-01	2.9E-05*	1.3E-17*
MASQ AA	11 ± 1.4	18 ± 10	16 ± 6.0	4.6E-01	4.4E-07*	1.8E-10*
PSS	9 ± 5.9	25 ± 7.1	24 ± 6.3	5.8E-01	2.9E-12*	1.1E-25*
CTQ	32 ± 7.5	33 ± 5.4	48 ± 14	4.3E-04*	7.4E-01	1.4E-13*
RRS	29 ± 8.4	56 ± 13	57 ± 12	8.5E-01	4.1E-14*	5.4E-29*
SHAPS	0 ± 1.8	5 ± 4.6	7 ± 4.4	3.7E-01	1.1E-08*	2.3E-18*
TEPS-A	45 ± 7.4	31 ± 13	33 ± 10	5.5E-01	6.3E-07*	1.9E-12*
TEPS-C	39 ± 6.5	28 ± 9.2	30 ± 9.6	5.5E-01	9.2E-06*	2.1E-08*
STICSA C	11 ± 2.4	29 ± 5.1	23 ± 6.0	3.3E-03*	9.8E-31*	5.7E-29*
STICSA S	12 ± 1.5	18 ± 8.0	18 ± 6.4	1.0E+00	1.3E-08*	2.2E-13*
Left Caudal Middle Frontal	12 ± 1.2	12 ± 0.9	11 ± 1.4	2.3E-02*	1.3E-01	7.1E-02
Right Caudal Middle Frontal	11 ± 1.6	12 ± 1.6	11 ± 1.7	3.5E-02*	7.9E-02	3.3E-01
Left Cuneus	5.1 ± 0.6	4.6 ± 0.5	5.1 ± 0.6	3.1E-02*	4.2E-02*	8.6E-01
Left Entorhinal	2.2 ± 0.4	2.7 ± 1.5	2.2 ± 0.5	4.0E-02*	7.6E-03*	5.8E-01
Right Superior Frontal	36 ± 2.6	37 ± 2.3	36 ± 3.0	6.5E-02	3.4E-02*	9.6E-01
Right Supramarginal	17 ± 1.6	16 ± 2.7	17 ± 2.1	4.7E-02*	6.4E-02	2.5E-01
Right Transverse Temporal	1.4 ± 0.2	1.3 ± 0.1	1.4 ± 0.2	1.0E-01	4.5E-02*	9.1E-01
Right Thalamus	6.1 ± 0.5	6.3 ± 0.2	6.3 ± 0.4	8.9E-01	1.7E-01	2.7E-02*

All imaging derived-volumetric measures showing significant differences between two cohorts are included CL 2 showed significantly higher CTQ scores than either CL 1 or healthy controls, whereas CL 1 was not different from healthy controls in this respect. No major differences in age, gender, BMI or age of onset were seen between the two MDD clusters. Bold font and asterisks indicates p-values < 0.05 based on a two-sided two-tailed student’s t-test.

**Table 5:** Canonical variables CV 1–3 and IV 1–3 for patient clusters CL1 and CL2.

7TID	Controls	Cluster 1	Cluster 2	CL 1 vs. CL 2	HC vs. CL 1	HC vs. CL 2
CV 1	-1.6 ± 0.5	0.3 ± 0.6	-1.6 ± 1.2	8.0E-06*	2.5E-18*	9.7E-01
CV 2	-5.7 ± 0.8	-5.3 ± 0.6	-6.8 ± 0.9	5.1E-06*	8.5E-02	6.0E-10*
CV 3	1.9 ± 0.8	0.9 ± 0.5	1.4 ± 1.2	1.1E-01	9.2E-05*	1.5E-02*
IV 1	-1.2 ± 0.6	0.4 ± 0.7	-1.2 ± 1.2	1.1E-04*	5.5E-13*	7.9E-01
IV 2	-27.1 ± 0.8	-26.7 ± 0.6	-28.1 ± 1.0	2.8E-05*	1.3E-01	3.0E-08*
IV 3	20.1 ± 0.9	19.1 ± 0.4	19.7 ± 1.1	9.6E-02	4.9E-04*	2.0E-02*

For a given canonical pair (i.e. CV 1 and IV 1), these represent linear combinations of clinical symptom scales and imaging-derived metrics, respectively, such that the canonical pairs are highly correlated. The two clusters showed significant differences in CV 1, CV 2 and the corresponding IV 1 & IV 2. The clusters were not significantly different in CV 3 and IV 3. Bold font and asterisks indicates p-values < 0.05 based on a two-sided two-tailed student’s t-test.

Between CL 1 and CL 2, five of the 87 imaging features considered, showed differences at the p < 0.05 level. These differences do not survive correction for multiple comparisons, however, and closely match the number of differences that would be expected from random chance alone. Likewise, any differences in imaging features between either cluster and healthy controls also do not survive multiple comparisons.

**Figure 6:**
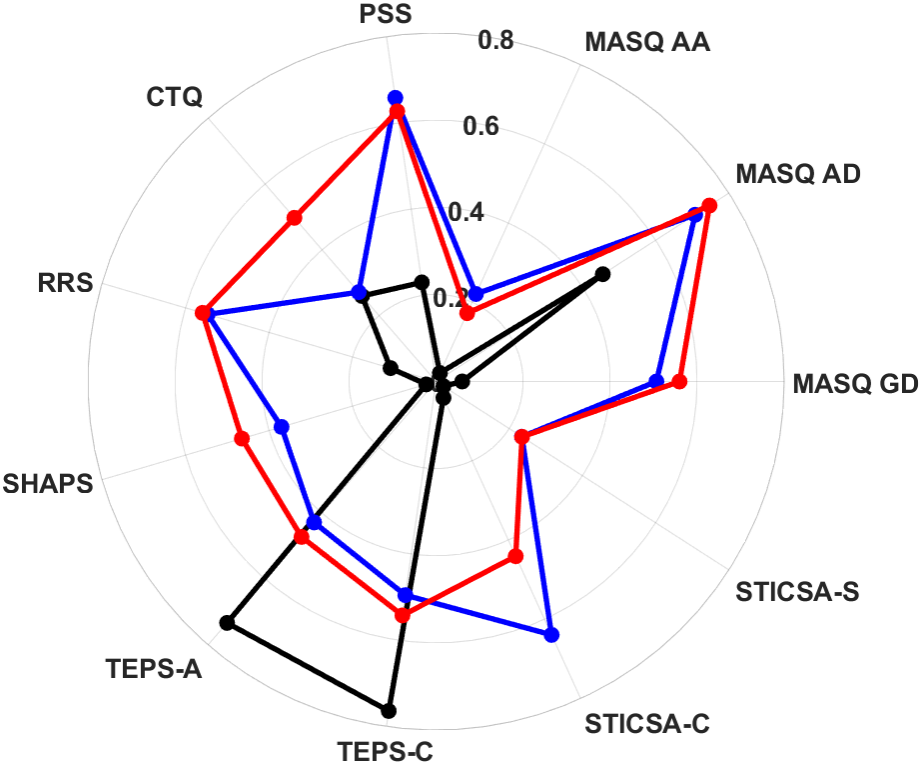
Radar chart showing symptom of derived MDD patient clusters and healthy controls. CL 1 is shown in blue, CL 2 in red and controls in black. The symptom severity scales are normalized such that 0 is the minimum individual observed value and 1 is the maximum individual observed value.

**Figure 6** shows a visual representation of the two MDD patient clusters in terms of clinical symptom severity. CL 1 is shown in blue, CL 2 is show in red, and healthy controls are included for comparison and shown in black. The data and statistical tests underlying the two clusters are listed quantitatively in **Table 4**. CL 1 represents a majority (53 out of 64) of the patients whose CTQ scores indicate child trauma measures not significantly different from healthy controls, but who differed significantly from controls amongst all other clinical scales. In particular, the anxiety-related symptom scale STICSA-C was significantly higher among CL 1 than both healthy controls and CL 2. The symptom severity scales for **Figure 6** are normalized such the maximum individual observation for each measure is equal to 1 and the minimum individual observation is equal to 0.

## Discussion

In this study, we introduce a data-driven, non-biased ML classification framework that integrates clinical symptom measures with high-resolution MRI-derived features. Using CCA and hierarchical clustering, the approach identifies distinct subtypes of MDD, offering a more objective and potentially predictive alternative to traditional methods. CCA yielded three axes of highly-correlated clinical-imaging phenotypes which were subsequently used to classify depression patients. These axes highlighted loading from both clinical and imaging components. CV 1 was most strongly correlated with IV 1 and showed loading from anhedonia related measures like TEPS-C and MASQ AD. The corresponding IV showed greatest loading from the brain stem. CV 2 also showed loading from the anhedonia-related variables TEPS-C and MASQ AD with greatest loading from the childhood trauma related measure CTQ. The related IV 2 showed loading from right frontal pole and both left and right precuneus. Both right amygdala and right hippocampus were among the top ten regions correlated with IV2. CV 3 showed loading from the stress and anxiety related measures (PSS, MASQ GD and STICSA) with the related IV 3 showing greatest loading from right superior temporal lobe, right insula and left medial orbitofrontal lobe. The frontal lobe has been extensively studied in depression. For example, Almeida et al. (30) demonstrated atrophy of right frontal lobe associated with late-onset depression and Tang et al. (31) found frontal lobe atrophy in depressed patients following stroke. Brainstem volume was found to be the most highly-correlated imaging feature to the CV 1 + IV 1 phenotype. The brainstem has gained interest in the study of depression as the site of the raphe nuclei, which regulate the release of serotonin, and are believed targeted in selective serotonin reuptake inhibitor (SSRI) category of antidepressants. Projections from the Raphe nucleus extend to the forebrain (32, 33) where they are believed to modulate mood, memory and sleep cycles.

The CV 2 + IV 2 phenotype showed high loading of its components from anhedonia measures and frontal lobe. This supports previous studies which have shown the frontal poles to be a region that activates more to happy stimuli than sad stimuli under fMRI (34, 35). A 2021 study (36) also implicated right frontal pole and right medial prefrontal complex with anhedonia as measured by the SHAPS, an important loading factor in CV 2. Childhood trauma, as measured by the CTQ, was shown to correlate with lower volumes of amygdala and hippocampus in healthy controls, yet higher volumes among patients with bipolar disorder (37). Furthermore, a 2023 study (38) investigating functional connectivity between amygdala and hippocampus in association with clinical symptoms of major depression found reduced functional connectivity between right amygdala and right precuneus in patients with childhood trauma. The same study found a significant negative correlation between functional connectivity between these regions and anhedonia. The CV 2 + IV 2 phenotype likewise associates strongly negative loading from anhedonia and childhood trauma measures with higher volumes of right amygdala and right precuneus.

Previous approaches to developing biological subtypes for depression, such as Drysdale et al. (21) and Tozzi et al. (39), were based on an fMRI network based approach. There is comparatively little literature attempting to derive these biological subtypes based on anatomical imaging. For example, a 2019 review of CCA for deriving biological subtypes mentions no studies using anatomical neuroimaging (40). The presented study leverages the greater sensitivity of ultrahigh field MRI towards deriving subtypes of depression based on high-resolution anatomical imaging and subsequent whole brain segmentation. The volumetric changes in depression assessed by anatomical MRI may present different effects of the disease than the altered functional connectivity assessed by fMRI, though future CCA analyses may combine multiple imaging modalities as these studies combine clinical symptom scales.

Significant differences in imaging features were not observed either between the two clusters of MDD patients, or between either clustered group and healthy controls. Instead, the imaging-derived metrics showing differences between clustered MDD patients and healthy controls were the imaging canonical variables IV 1 and IV 2. Clustering patients along the canonical variables is not intended to identify individual clinical or imaging metrics which stratify major depression. Instead, the canonical variables establish clinical-imaging subtypes of depression which may stratify patients with MDD better than the individual component features could.

Childhood trauma, as represented by the CTQ, was a major loading factor in two of the three canonical variables (CV 1 and CV 2) and also a differentiator between the two patient clusters. CL 1 showed CTQ scores not significantly different from healthy controls, while CL 2 showed CTQ scores significantly higher than both CL 1 and healthy controls. This is particularly notable as CL 1 consisted of 53 MDD patients, compared to only 11 patients in CL 2. The hierarchical clustering therefore effectively separated the small minority of MDD patients who experienced childhood trauma at rates significantly above controls from all remaining MDD patients. This suggests presence of childhood trauma may be an important differentiator between cohorts of MDD patients with otherwise similar clinical characteristics.

Large-scale imaging acquisition at 7T is complicated, as some volunteers self-exclude based on claustrophobic anxiety triggered by the long, narrow bore and others are subject by stringent exclusion criteria around metallic objects like piercings and copper IUDs not typically tested for safety at higher field strengths. Automatic imaging segmentation algorithms may show systemic differences at different field strengths, and studies relying exclusively on imaging data acquired at ultrahigh field will be typically limited in sample size. This sample size limitation in turn affects optimal cluster size analysis and precludes the classification of such a limited cohort into many biological subtypes and reduces the power of statistical analysis between the derived clusters. As 7T scanners gain FDA approval and become more prevalent, acquisition of larger scale datasets may become more feasible, facilitating more granular classification strategies like those currently seen with 3T MRI.

In summary, we present a novel ML framework for analyzing multi-modal imaging data and clinical scales to classify major depression. Acquiring more data may facilitate greater precision in the correlation analysis and potentially yield more and better-defined clinical-imaging phenotypes. While subject to the limitations of scan resolution, imaging-derived metrics are more reproducible and less subject to reader bias than classifications based on observed clinical characteristics alone, and thus may play a role in the characterization of this complex disease.

## Data Availability

The datasets presented in this study can be found in online repositories. The names of the repository/repositories and accession number(s) can be found below: De-identified T1 imaging used in the data was shared with the NIMH Data Archive.

## References

[1] Kessler RC Berglund P Demler O Jin R Merikangas KR Walters EE . Lifetime prevalence and age-of-onset distributions of DSM-IV disorders in the National Comorbidity Survey Replication. Arch Gen Psychiatry. (2005) 62:593–602. doi: 10.1001/archpsyc.62.6.593, PMID: 15939837

[2] Kessler RC Berglund P Demler O Jin R Koretz D Merikangas KR . The epidemiology of major depressive disorder: results from the National Comorbidity Survey Replication (NCS-R). Jama. (2003) 289:3095–105. doi: 10.1001/jama.289.23.3095, PMID: 12813115

[3] Spaner D Bland R Newman S . Major depressive disorder. Acta Psychiatrica Scandinavica. (1994) 89:7–15. doi: 10.1111/j.1600-0447.1994.tb05786.x, PMID: 8178688

[4] Greenberg PE Fournier A-A Sisitsky T Simes M Berman R Koenigsberg SH . The economic burden of adults with major depressive disorder in the United States (2010 and 2018). Pharmacoeconomics. (2021) p:1–13. doi: 10.1007/s40273-021-01019-4, PMID: 33950419 PMC8097130

[5] Fava M . Diagnosis and definition of treatment-resistant depression. Biol Psychiatry. (2003) 53:649–59. doi: 10.1016/S0006-3223(03)00231-2, PMID: 12706951

[6] Insel TR Cuthbert BN . Brain disorders? precisely. Science. (2015) 348:499–500. doi: 10.1126/science.aab2358, PMID: 25931539

[7] Dinga R Schmaal L Penninx B Tol MJv Veltman DJ Velzen Lv . Evaluating the evidence for biotypes of depression: attempted replication of Drysdale et al., 2017. . bioRxiv. (2018) p:416321. doi: 10.1101/416321

[8] Verma G Balchandani P . Ultrahigh field MR neuroimaging. Topics Magnetic Resonance Imaging. (2019) 28:137–44. doi: 10.1097/RMR.0000000000000210, PMID: 31188272 PMC6784841

[9] Jones SE Lee J Law M . Neuroimaging at 3T vs 7T: is it really worth it? Magnetic Resonance Imaging Clinics. (2021) 29:1–12. doi: 10.1016/j.mric.2020.09.001, PMID: 33237010

[10] Van der Kolk AG Hendrikse J Zwanenburg JJ Visser F Luijten PR . Clinical applications of 7 T MRI in the brain. Eur J Radiol. (2013) 82:708–18. doi: 10.1016/j.ejrad.2011.07.007, PMID: 21937178

[11] Davidson J Turnbull CD Strickland R Miller R Graves K . The Montgomery-Åsberg Depression Scale: reliability and validity. Acta psychiatrica scandinavica. (1986) 73:544–8. doi: 10.1111/j.1600-0447.1986.tb02723.x, PMID: 3751660

[12] Watson D Clark LA . Mood and anxiety symptom questionnaire. J Behav Ther Exp Psychiatry. (1991). doi: 10.1037/t13679-000, PMID: 41770175

[13] Cohen S Kamarck T Mermelstein R . Perceived stress scale. Measuring stress: A guide Health Soc scientists. (1994) 10:1–2. doi: 10.2307/2136404, PMID: 39964225

[14] Bernstein DP Fink L . Childhood Trauma Questionnaire: a retrospective self-report manual. The Psychological Corporation, San Antonio, TX. (1998).

[15] Roelofs J Muris P Huibers M Peeters F Arntz A . On the measurement of rumination: A psychometric evaluation of the ruminative response scale and the rumination on sadness scale in undergraduates. J Behav Ther Exp Psychiatry. (2006) 37:299–313. doi: 10.1016/j.jbtep.2006.03.002, PMID: 16678121

[16] Snaith RP Hamilton M Morley S Humayan A Hargreaves D Trigwell P . A scale for the assessment of hedonic tone the Snaith–Hamilton Pleasure Scale. Br J Psychiatry. (1995) 167:99–103. doi: 10.1192/bjp.167.1.99, PMID: 7551619

[17] Gard DE Gard MG Kring AM John OP . Anticipatory and consummatory components of the experience of pleasure: a scale development study. J Res Pers. (2006) 40:1086–102. doi: 10.1016/j.jrp.2005.11.001, PMID: 41853590

[18] Grös DF Antony MM Simms LJ McCabe RE . Psychometric properties of the state-trait inventory for cognitive and somatic anxiety (STICSA): comparison to the state-trait anxiety inventory (STAI). psychol Assess. (2007) 19:369. doi: 10.1037/1040-3590.19.4.369, PMID: 18085930

[19] Fischl B . FreeSurfer. Neuroimage. (2012) 62:774–81. doi: 10.1016/j.neuroimage.2012.01.021, PMID: 22248573 PMC3685476

[20] Mihalik A Adams RA Huys Q . Canonical correlation analysis for identifying biotypes of depression. Biol Psychiatry: Cogn Neurosci Neuroimaging. (2020) 5:478–80. doi: 10.1016/j.bpsc.2020.02.002, PMID: 32224000

[21] Drysdale AT Grosenick L Downar J Dunlop K Mansouri F Meng Y . Resting-state connectivity biomarkers define neurophysiological subtypes of depression. Nat Med. (2017) 23:28–38. doi: 10.1038/nm.4246, PMID: 27918562 PMC5624035

[22] Zhang T Tang X Li H Woodberry KA Kline ER Xu L . Clinical subtypes that predict conversion to psychosis: a canonical correlation analysis study from the ShangHai At Risk for Psychosis program. Aust New Z J Psychiatry. (2020) 54:482–95. doi: 10.1177/0004867419872248, PMID: 31486343

[23] Brusov O Kolyaskina G Kaleda V Sekirina T Kushner S Vasilieva E . Use of canonical correfslation analysis to evaluate the strength of relationships between clinical and biological parameters. Neurosci Behav Physiol. (2011) 41:375–83. doi: 10.1007/s11055-011-9425-6, PMID: 41853694

[24] Keogh E Reidy J . Exploring the factor structure of the Mood and Anxiety Symptom Questionnaire (MASQ). J Pers Assess. (2000) 74:106–25. doi: 10.1207/S15327752JPA740108, PMID: 10779936

[25] Rousseeuw PJ . Silhouettes: a graphical aid to the interpretation and validation of cluster analysis. J Comput Appl mathematics. (1987) 20:53–65. doi: 10.1016/0377-0427(87)90125-7

[26] Caliński T Harabasz J . A dendrite method for cluster analysis. Commun Statistics-theory Methods. (1974) 3:1–27. doi: 10.1080/03610927408827101, PMID: 41799851

[27] Davies DL Bouldin DW . A cluster separation measure Vol. 2). New York, NY, United States: IEEE transactions on pattern analysis and machine intelligence (1979) p. 224–7. 21868852

[28] Von Luxburg U . A tutorial on spectral clustering. . Stat computing. (2007) 17:395–416. doi: 10.1007/s11222-007-9033-z, PMID: 41853694

[29] D’agostino R Pearson ES . Tests for departure from normality. Empirical results for the distributions of b 2 and√ b. Biometrika. (1973) 60:613–22. doi: 10.1016/j.neuroimage.2012.01.021, PMID: 22248573 PMC3685476

[30] Almeida O Burton E Ferrier N McKeith I O'BRIEN JT . Depression with late onset is associated with right frontal lobe atrophy. psychol Med. (2003) 33:675–81. doi: 10.1017/S003329170300758X, PMID: 12785469

[31] Tang W Chen YK Lu JY Mok VC Chu WC Ungvari GS . Frontal lobe atrophy in depression after stroke. Stroke Res Treat. (2013) 2013:424769. doi: 10.1155/2013/424769, PMID: 23533960 PMC3596905

[32] Walker EP Tadi P . Neuroanatomy, nucleus raphe. In: StatPearls. Treasure Island, FL, USA: StatPearls Publishing LLC (2023). 31335079

[33] Lee H-Y Tae WS Yoon H-K Lee B-T Paik J-W Son K-R . Demonstration of decreased gray matter concentration in the midbrain encompassing the dorsal raphe nucleus and the limbic subcortical regions in major depressive disorder: an optimized voxel-based morphometry study. J Affect Disord. (2011) 133:128–36. doi: 10.1016/j.jad.2011.04.006, PMID: 21546094

[34] Keedwell PA Andrew C Williams SC Brammer MJ Phillips ML . The neural correlates of anhedonia in major depressive disorder. Biol Psychiatry. (2005) 58:843–53. doi: 10.1016/j.biopsych.2005.05.019, PMID: 16043128

[35] Keedwell PA Andrew C Williams SC Brammer MJ Phillips ML . A double dissociation of ventromedial prefrontal cortical responses to sad and happy stimuli in depressed and healthy individuals. Biol Psychiatry. (2005) 58:495–503. doi: 10.1016/j.biopsych.2005.04.035, PMID: 15993859

[36] Shaw SR El-Omar H Roquet D Hodges JR Piguet O Ahmed RM . Uncovering the prevalence and neural substrates of anhedonia in frontotemporal dementia. Brain. (2021) 144:1551–64. doi: 10.1093/brain/awab032, PMID: 33843983

[37] Janiri D Sani G Rossi PD Piras F Iorio M Banaj N . Amygdala and hippocampus volumes are differently affected by childhood trauma in patients with bipolar disorders and healthy controls. Bipolar Disord. (2017) 19:353–62. doi: 10.1111/bdi.12516, PMID: 28699182

[38] Fan J Gao F Wang X Liu Q Xia J Han Y . Right amygdala–right precuneus connectivity is associated with childhood trauma in major depression patients and healthy controls. Soc Cogn Affect Neurosci. (2023) 18:nsac064. doi: 10.1093/scan/nsac064, PMID: 36639930 PMC10036873

[39] Tozzi L Zhang X Pines A Olmsted AM Zhai ES Anene ET . Personalized brain circuit scores identify clinically distinct biotypes in depression and anxiety. Nat Med. (2024) 30:2076–87. doi: 10.1038/s41591-024-03057-9, PMID: 38886626 PMC11271415

[40] Beijers L Wardenaar KJ van Loo HM Schoevers RA . Data-driven biological subtypes of depression: systematic review of biological approaches to depression subtyping. Mol Psychiatry. (2019) 24:888–900. doi: 10.1038/s41380-019-0385-5, PMID: 30824865

