# Supplementary material for: Machine learning framework for depression subtype grouping: integrating high-resolution imaging and clinical symptom analysis via correlation and clustering
Source: Front Psychiatry. 2026 Mar 24;17:1747824. doi: 10.3389/fpsyt.2026.1747824 (PMC13055587; doi:10.3389/fpsyt.2026.1747824)
Supplement: Supplementary file 1 [file DataSheet1.docx]

MDD Machine Learning Supplementary Data

The combination of canonical correlation analysis and hierarchical clustering can be iterated through multiple analysis strategies, in particular along the following axes:

1. Clustering on MDD patients only or a combination of patients + controls.
2. Clustering on clinically-loaded variables CV 1-3 or imaging feature loaded variables IV 1-3.
3. Pre-scan normalization
4. Alternative clustering on the combination of both MDD patients and controls (total n=131) led to two imbalanced clusters of n=9 and n=122. The smaller cluster was made of predominantly young, female MDD patients. Among the 9 subjects in the first cluster, one was a male MDD patient, one was a female healthy control, and the remaining seven were female MDD patients. Their mean age was 27.7 years. By comparison, the larger group consisted of 56 MDD patients, 66 controls, 56 female subjects and 66 male with a total mean age of 34.1 years. When the combined cohort of 131 subjects was split into 3 clusters, the same 9 subjects formed the first cluster. The remaining 122 subjects were split into clusters of n=59 (of which 51 were controls, 31 male, mean age 34.7 years) and n=63 (of which 15 were controls, 35 male, mean age 33.6 years). These clusters thus effectively separated the subjects into groups of predominantly (76.2%) MDD patients and predominantly (86.4%) controls that were otherwise similar in age and sex composition.
5. Though IV 1-3 were highly correlated with CV 1-3, small differences in clustering results were observed between them. Clustering on the imaging-derived variables resulted in cluster sizes of n = 51 for cluster 1 and n = 13 for cluster 2. Of the 64 total MDD patients, 60 remained in the same cluster, with three subjects shifting from cluster 1 to cluster 2 and one subject shifting in reverse when the clustering was performed on IV 1-3. The two clusters retained significant differences in CTQ and STICSA-C clinical measures, and additionally showed a difference in TEPS-C with cluster 1 averaging a 30.5 score and cluster 2 averaging 24.4 in the measure (p = 0.035). Six imaging features showed differences between the two clusters, yet this is comparable to the number expected by random chance, and none would survive FDR-adjustment for multiple comparisons.

Clinical measure loading factors in canonical variables CV 1-3

|  | CV1 | CV2 | CV3 |
| --- | --- | --- | --- |
| masq_gd_score | -0.06215 | -0.35333 | -0.46154 |
| masq_ad_score | 0.279438 | -0.61263 | -0.08161 |
| masq_aa_score | -0.00983 | -0.34712 | -0.29777 |
| pss_score | 0.113799 | -0.44403 | -0.59087 |
| ctq_total | -0.26665 | -0.66853 | -0.17659 |
| rrs_total | 0.096722 | -0.4059 | -0.20449 |
| shaps_score_2 | 0.274612 | -0.42615 | 0.011294 |
| TEPS-A | -0.22048 | 0.215065 | 0.100954 |
| TEPS-C | -0.36938 | 0.346753 | 0.092826 |
| sticsa_cognitive | 0.194503 | -0.25664 | -0.42346 |
| sticsa_somatic | -0.24078 | -0.27499 | -0.21052 |

Imaging-derived measure loading factors in canonical variables CV 1-3

| Region | CV1 | CV2 | CV3 |
| --- | --- | --- | --- |
| Rsuperiortemporal | 0.166415 | 0.013158 | 0.277584 |
| Rinsula | 0.129636 | 0.000654 | 0.190771 |
| Lmedialorbitofrontal | 0.029947 | 0.077718 | 0.183538 |
| Rbankssts | 0.121582 | 0.066963 | 0.181235 |
| Rlateraloccipital | 0.05472 | 0.05686 | 0.175359 |
| Rfusiform | 0.257574 | 0.022173 | 0.174406 |
| Rrostralanteriorcingulate | 0.038541 | 0.057801 | 0.169893 |
| Lpericalcarine | 0.204162 | 0.114292 | 0.158533 |
| Rparstriangularis | 0.00803 | 0.001807 | 0.154916 |
| Lparahippocampal | 0.051275 | 0.049076 | 0.153295 |
| Rsupramarginal | 0.026525 | 0.099105 | 0.152745 |
| Rposteriorcingulate | 0.105567 | 0.150243 | 0.146755 |
| N_Right-Thalamus-Proper | 0.0746 | 0.009089 | 0.142807 |
| Ltransversetemporal | 0.029637 | 0.141501 | 0.1425 |
| Linferiorparietal | 0.106353 | 0.031714 | 0.127692 |
| Lsuperiortemporal | 0.154448 | 0.02895 | 0.126646 |
| Lparacentral | 0.079701 | 0.115743 | 0.123879 |
| Lmiddletemporal | 0.155416 | 0.073039 | 0.122333 |
| Lparsorbitalis | 0.125507 | 0.097008 | 0.117177 |
| N_Left-Amygdala | 0.005788 | 0.032556 | 0.116677 |
| Lparsopercularis | 0.087359 | 0.027091 | 0.109258 |
| Lcuneus | 0.26283 | 0.067757 | 0.108776 |
| Rtransversetemporal | 0.094363 | 0.064003 | 0.101962 |
| N_Right-VentralDC | 0.097221 | 0.075067 | 0.101006 |
| Rsuperiorparietal | 0.181099 | 0.038618 | 0.096999 |
| Rmiddletemporal | 0.068238 | 0.078777 | 0.09289 |
| Linferiortemporal | 0.058806 | 0.006107 | 0.089583 |
| Lposteriorcingulate | 0.036169 | 0.026191 | 0.087398 |
| N_Left-Pallidum | 0.093714 | 0.164297 | 0.086068 |
| Rpericalcarine | 0.219519 | 0.097602 | 0.085674 |
| Rcuneus | 0.211447 | 0.063866 | 0.081543 |
| N_Right-Caudate | 0.156333 | 0.029372 | 0.081038 |
| Rlateralorbitofrontal | 0.151204 | 0.030045 | 0.080284 |
| N_Left-Caudate | 0.171227 | 0.023438 | 0.078299 |
| N_Left-Thalamus-Proper | 0.001231 | 0.025022 | 0.075703 |
| Linsula | 0.059652 | 0.013115 | 0.075477 |
| Rsuperiorfrontal | 0.090266 | 0.010232 | 0.0754 |
| N_Left-choroid-plexus | 0.122925 | 0.019962 | 0.074802 |
| Listhmuscingulate | 0.028989 | 0.019898 | 0.072295 |
| Lsupramarginal | 0.177138 | 0.097543 | 0.070215 |
| Lpostcentral | 0.113112 | 0.019388 | 0.06304 |
| Lbankssts | 0.108075 | 0.023318 | 0.061661 |
| Rprecuneus | 0.172574 | 0.220808 | 0.0616 |
| Lcaudalmiddlefrontal | 0.032505 | 0.100832 | 0.05958 |
| Llingual | 0.006699 | 0.065812 | 0.058996 |
| Rparahippocampal | 0.036242 | 0.004465 | 0.0582 |
| Lfusiform | 0.018035 | 0.104879 | 0.05519 |
| Lcaudalanteriorcingulate | 0.063057 | 0.03436 | 0.054031 |
| Lprecuneus | 0.22693 | 0.219688 | 0.053899 |
| Lsuperiorfrontal | 0.150564 | 0.016103 | 0.05272 |
| Rinferiorparietal | 0.070746 | 0.055368 | 0.052138 |
| Rfrontalpole | 0.042697 | 0.301424 | 0.051673 |
| Brain-Stem | 0.308078 | 0.079582 | 0.051414 |
| Rcaudalanteriorcingulate | 0.01806 | 0.13662 | 0.051245 |
| Lfrontalpole | 0.036022 | 0.006087 | 0.046919 |
| Lsuperiorparietal | 0.147301 | 0.094665 | 0.04392 |
| Rparacentral | 0.032252 | 0.095962 | 0.043902 |
| Rentorhinal | 0.031718 | 0.135722 | 0.043695 |
| Lrostralmiddlefrontal | 0.044235 | 0.140762 | 0.041026 |
| Rrostralmiddlefrontal | 0.125086 | 0.141475 | 0.040979 |
| N_Right-Pallidum | 0.146037 | 0.013514 | 0.036225 |
| Rtemporalpole | 0.013411 | 0.042339 | 0.034507 |
| Rlingual | 0.239946 | 0.099736 | 0.034295 |
| Rinferiortemporal | 0.012872 | 0.010585 | 0.034155 |
| Rprecentral | 0.028247 | 0.082215 | 0.033168 |
| Rparsopercularis | 0.003239 | 0.105926 | 0.031057 |
| **N_Right-Hippocampus** | **0.103178** | **0.165535** | **0.030687** |
| N_Right-Amygdala | 0.000221 | 0.213558 | 0.028785 |
| N_Left-Putamen | 0.078604 | 0.092252 | 0.028296 |
| Rmedialorbitofrontal | 0.095461 | 0.036663 | 0.028248 |
| Risthmuscingulate | 0.031173 | 0.033465 | 0.027026 |
| N_Left-AN_CCumbens-area | 0.002281 | 0.079923 | 0.026394 |
| Rcaudalmiddlefrontal | 0.154602 | 0.191496 | 0.022869 |
| Rpostcentral | 0.060095 | 0.032998 | 0.022575 |
| Llateraloccipital | 0.242592 | 0.18722 | 0.020779 |
| Ltemporalpole | 0.001167 | 0.064296 | 0.020583 |
| Lrostralanteriorcingulate | 0.010569 | 0.078105 | 0.016419 |
| N_Right-Putamen | 0.093772 | 0.052809 | 0.015936 |
| **N_Left-Hippocampus** | **0.063892** | **0.101797** | **0.015501** |
| N_Right-AN_CCumbens-area | 0.030705 | 0.057658 | 0.012589 |
| N_Left-VentralDC | 0.175801 | 0.072603 | 0.012381 |
| Llateralorbitofrontal | 0.144298 | 0.016209 | 0.011744 |
| Rparsorbitalis | 0.007677 | 0.026223 | 0.00979 |
| N_Right-choroid-plexus | 0.173936 | 0.040603 | 0.009587 |
| Lprecentral | 0.025217 | 0.014857 | 0.009458 |
| Lentorhinal | 0.226475 | 0.027821 | 0.008379 |
| Lparstriangularis | 0.124673 | 0.059843 | 0.005681 |
